# Reliability of the MOCART score: a systematic review

**DOI:** 10.1186/s10195-021-00603-w

**Published:** 2021-10-06

**Authors:** Filippo Migliorini, Nicola Maffulli, Jörg Eschweiler, Arne Driessen, Markus Tingart, Alice Baroncini

**Affiliations:** 1 Department of Orthopaedic, Trauma, and Reconstructive Surgery, RWTH University Hospital Aachen, Aachen, Germany; 2 Department of Medicine, Surgery and Dentistry, University of Salerno, Via S. Allende, 84081 Baronissi, SA Italy; 3 School of Pharmacy and Bioengineering, Keele University School of Medicine, Thornburrow Drive, Stoke on Trent, England, UK; 4 Queen Mary University of London, Barts and the London School of Medicine and Dentistry, Centre for Sports and Exercise Medicine, Mile End Hospital, 275 Bancroft Road, London, E1 4DG England, UK

**Keywords:** Chondral defect, Knee, Talus, MOCART

## Abstract

**Background:**

The present systematic review analysed the available literature to assess reliability of the Magnetic Resonance Observation of Cartilage Repair Tissue (MOCART) score in the evaluation of knee and ankle osteochondral lesions.

**Methods:**

All the studies using the MOCART score for knee and/or talus chondral defects were accessed in March 2021. A multivariate analysis was performed to assess associations between the MOCART score at last follow-up and data of patients at baseline, clinical scores and complications. A multiple linear model regression analysis was used.

**Results:**

The MOCART score evidenced no association with patient age (*P* = 0.6), sex (*P* = 0.1), body mass index (*P* = 0.06), defect size (*P* = 0.9), prior length of symptoms (*P* = 0.9) or visual analogue scale (*P* = 0.07). For chondral defects of the knee, no statistically significant association was found between the MOCART score and the International Knee Documentation Committee (*P* = 0.9) and with the Lysholm Knee Scoring Scales (*P* = 0.2), Tegner Activity Scale (*P* = 0.2), visual analogue scale *P* = 0.07), rate of failure (*P* = 0.2) and revision (*P* = 0.9). For chondral defect of the talus, no statistically significant associations were found between the MOCART score and the American Orthopedic Foot and Ankle Score (*P* = 0.3), Tegner Activity Scale (*P* = 0.4), visual analogue scale (*P* = 0.1), rate of failure (*P* = 0.1) and revision (*P* = 0.7).

**Conclusion:**

The MOCART score demonstrated no association with patient characteristics and with the surgical outcome in patients who underwent surgical management for knee and talus chondral defects.

**Level of evidence:**

Level IV.

## Introduction

Acute injuries, repeated strains or joint instability can produce osteochondral lesions (OL), with damage to the hyaline cartilage of the joint and to the subchondral bone [1, 2]. Typically, these lesions lead to a decrease in daily activities from pain on weight bearing and exercise [3]. Several surgical techniques are available for the operative management of OL of the knee and ankle: it necessary to have reliable evaluation tools to compare the outcomes of the different techniques and offer clinically meaningful feedback to patients. Arthroscopy remains the gold standard for the evaluation of the cartilage after treatment [4], but non-invasive follow-up methods are required for post-operative assessment. Plain radiography and computed tomography are inadequate for the analysis of cartilage layers, and the ionizing radiations required are of concern [5]. Magnetic resonance imaging (MRI), on the other hand, allows a detailed analysis of the cartilage and does not require ionizing radiation [6], and has thus become a widespread tool for post-operative evaluation of the outcome of OL [7]. To assess and quantify possible changes after treatment, Marlovits and colleagues introduced the Magnetic Resonance Observation of Cartilage Repair Tissue (MOCART) score [8]. However, its role in the evaluation of the treated cartilage and its clinical value are still debated [1, 2, 9, 10]. The MOCART Score analyses different MRI variables that should correlate with the success of the operative management of OL lesions. These include degree of repair and filling of the OL, integration to border zone, surface, structure and signal intensity of the repair tissue, aspect of the subchondral lamina and bone and presence of adhesions or effusion (CIT).

The present systematic review analysed the available literature regarding the use of the MOCART score in the evaluation of osteochondral lesions of the knee and ankle. We wished to ascertain possible associations between the MOCART and other evaluation tools, which would highlight the association of the MOCART score with clinical outcomes, and support the use of this score for clinical use.

## Methods

### Search strategy

This systematic review was conducted according to the Preferred Reporting Items for Systematic Reviews and Meta-Analyses: the PRISMA statement [11]. The PICO framework was followed:

- P (Problem): knee and talus chondral defect;
- I (Intervention): surgical management;
- C (Control): MOCART score at last follow-up;
- O (Outcomes): clinical scores and complications.

### Data source

The literature search was conducted independently by two authors (**;**). In June 2021, the following databases were accessed: PubMed, Google scholar, Embase and Scopus with no time constrains. The following keywords were used in combination using the Boolean operators AND/OR: *chondral, cartilage, articular, damage, defect, injury, chondropathy, knee, pain, matrix-induced, periosteal, periosteum, collagen, autologous, chondrocyte, transplantation, implantation, MFX, microfractures, mosaicplasty, mACI, cACI, pACI, AMIC, OAT, osteochondral transplantation, allograft, autograft, membrane, therapy, management, surgery, outcomes, revision, hypertrophy, failure.* The resulting articles were screened by the same authors. The full text of the articles of interest were accessed. The bibliography of the full-text articles was also screened. Disagreements were debated and solved by a third author (**).

### Eligibility criteria

All the studies using the MOCART score for knee and/or talus chondral defects were accessed. Given the authors language abilities, articles in English, German, Italian, French and Spanish were eligible. Studies with level I–IV of evidence, according to Oxford Centre of Evidence-Based Medicine [12], were considered. Studies which reported data on patients with end-stage joint degeneration were not considered. Abstracts, reviews, comments, editorials and opinions were non considered. Animals, biomechanics or in vitro studies were not considered. Only studies which clearly stated the nature of the surgical intervention were included. Only articles reporting quantitative data under the outcomes of interest were considered for inclusion. Missing data under the outcomes of interest warranted the exclusion from this study.

### Data extraction

Data extraction was performed separately by two authors (**;**). Data concerning author, year, journal, type of study and length of the follow-up was extracted. Data of the MOCART score at last follow-up was collected. The following data at baseline was collected: number of samples with related mean body mass index (BMI) and age, duration of symptoms, percentage of female, size of the defect. Data concerning the following scores at last follow-up were retrieved according to their localisation (knee and talus): visual analogue scale (VAS), American Orthopedic Foot and Ankle Score (AOFAS) [13], Tegner Activity Scale [14], Lysholm Knee Scoring Scale [15], and International Knee Documentation Committee (IKDC) [16] scores. Data on complications (rate of hypertrophy, failure and revision) were also retrieved.

### Methodology quality assessment

The methodological quality assessment was performed by two independent authors (**;**). The risk of bias graph tool of the Review Manager Software (The Nordic Cochrane Collaboration, Copenhagen) was used. The following risk of bias were evaluated: selection, detection, attrition and other source of bias.

### Statistical analyses

All statistical analyses were performed by one author (F.M.) using the software STATA/MP 14.1 (StataCorp, College Station, TX). The Shapiro–Wilk test was performed to investigate data distribution. For normal data, mean and standard deviation were calculated. For non-parametric data, median and interquartile range were calculated. The Student *t*-test was used to assess significance for parametric data, while the Mann–Whitney *U*-test for non-parametric variables. Values of *P* < 0.05 considered statistically significant. A multivariate analysis was performed to assess associations between the values of the MOCART score at last follow-up and data of patients at baseline, clinical scores at last follow-up and the rate of complications. A multiple linear model regression analysis through the Pearson product-moment correlation coefficient ($r$) was used. The Cauchy–Schwarz formula was used for inequality: +1 is considered as positive linear correlation, while −1 is considered a negative one. Values of 0.1 < |$r$| < 0.3, 0.3 < |$r$| < 0.5, and |$r$| > 0.5 were considered to have weak, moderate and strong correlation, respectively. The overall significance was performed through the *χ*^2^ test, with values of *P* < 0.05 considered statistically significant.

## Results

### Search result

**Fig. 1:**
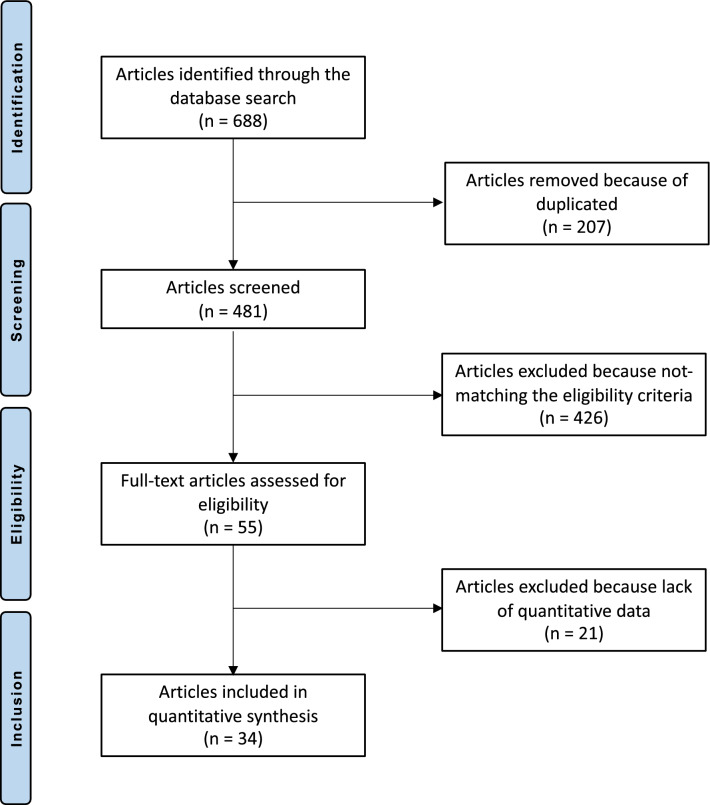
Flow chart of the literature search

The literature search identified 688 articles. Of them, 207 were duplicates. A further 481 articles were excluded as they did not match the eligibility criteria: not reporting data over the MOCART score (*N* = 301), not focusing on knee or ankle (*N* = 22), study design (*N* = 92), not reporting quantitative data under the outcomes of interest (*N* = 21), other (*N* = 9), language limitations (*N* = 2). This left 34 articles for the present study. The literature search results are shown in Fig. 1.

### Methodological quality assessment

**Fig. 2:**
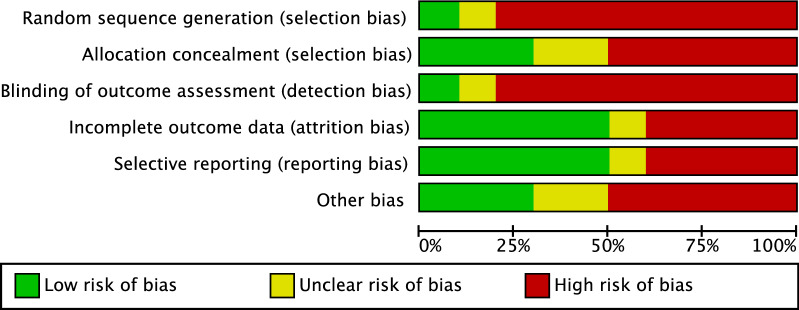
Methodological quality assessment

Given the limited number of randomised clinical studies (2 of 34) and the great number of retrospective studies (21 of 34), the risk of selection bias was moderate. The risk of detection bias was high, since most of studies lacked of blinding. The risk of attrition and reporting bias were estimated as moderate, as was the risk of other bias. In conclusion, the overall review authors’ judgements about each risk of bias item scored moderate, attesting to this study fair methodological assessment. The risk of bias graph is shown in Fig. 2.

### Patient demographics

Data from 1017 procedures were retrieved. The mean duration of symptoms before the index surgery was 30.1 ± 17.7 months. Forty percent (407 of 1017) were women. The mean age of the patients was 34.6 ± 6.1 years, the mean BMI was 25.7 ± 1.7 kg/m^2^. The mean defect size was 2.8 ± 2 cm^2^. The median follow-up was 37.1 (24 to 59.9) months. Generalities and demographic of the study are presented in Table 1.

**Table 1:** Generalities and patients baseline of the included studies

Author, year	Journal	Design	Follow-up (months)	Place	Type of treatment	Procedures (*n*)	Female (*%*)	Mean age
Albano et al. 2017 [21]	*BMC Musculos Dis*	Retrospective	30.0	Talus	AMIC	16	50.0	42.6
Anders et al. 2012 [22]	*Int Orthop*	Prospective	63.5	Talus	MACI	22	22.7	23.9
Apprich et al. 2012 [23]	*Osteoarthritis Cartilage*	Retrospective	48.0	Talus	MACT	10	60.0	31.0
			59.6	Talus	MFX	10	40.0	32.4
Astur et al. 2018 [24]	*Rev Bras Orthop*	Prospective	12.0	Knee	AMIC	7	14.3	37.2
Aurich et al. 2010 [25]	*Am J Sports Med*	Retrospective	24.5	Talus	MACI	19	27.8	29.2
Baumfeld et al. 2018 [26]	*Foot*	Retrospective	10.8	Talus	AMIC	17	47.1	37.5
Becher et al. 2015 [27]	*Arch Orthop Trauma Surg*	Prospective	21.0	Knee	MFX	5	40.0	27.0
DeSandis et al. 2018 [28]	*J Foot Ankle Surg*	Retrospective	16.7	Talus	Allo-transplantation	46	54.4	37.6
Dhollander et al. 2012 [29]	*Knee Surg Sports Traumatol Arthrosc*	Prospective	36.0	Knee	cACI	32	31.0	30.0
Di Cave et al. 2017 [30]	*The Foot*	Retrospective	90.0	Talus	Scaffold	12	25.0	38.6
Galla et al. 2018 [31]	*Knee Surg Sports Traumatol Arthrosc*	Retrospective	33.5	Talus	AMIC	23	34.8	35.6
Gottschalk et al. 2017 [32]	*J Foot Ankle Surg*	Retrospective	60.0	Talus	AMIC	21	38.1	37.0
Halem et al. 2014 [33]	*Am J Sports Med*	Retrospective	93.0	Talus	OAT	14	50.0	42.8
			85.3	Talus	OAT	28	39.3	44.1
Hoburg et al. 2019 [34]	*Orthop J Sports Med*	Prospective	63.0	Knee	mACI	29	48.0	16.0
			48.0	Knee	mACI	42	29.0	27.0
Karnovski et al. 2018 [35]	*Foot Ankle Int*	Retrospective	38.2	Talus	MFX	30	50.0	37.7
			19.4	Talus	Allo- transplantation	20	65.0	36.6
Koh et al. 2016 [36]	*Arthroscopy*	Prospective, Randomised	27.0	Knee	MFX	40	65.0	38.0
				Knee	MFX	40	60.0	39.0
Kubosch et al. 2015 [9]	*Int Orthop*	Retrospective	39.5	Talus	AMIC	17	47.1	38.8
Magnan et al. 2012 [37]	*Advance Orthop*	Retrospective	45.0	Talus	MACI	30	50.0	28.9
Marlovits et al. 2012 [38]	*Am J Sports Med*	Prospective	60.0	Knee	mACI	24	12.0	35.0
Niemeyer et al. 2013 [39]	*Am J Sports Med*	Prospective	131.0	Knee	pACI	70	64.0	33.0
Ogura et al. 2019 [40]	*Orthop J Sports Med*	Prospective	24.0	Knee	pACI, cACI	15	20.0	31.0
Perdisa et al. 2017 [41]	*Am J Sports Med*	Prospective	24.0	Knee	Scaffold	34	47.0	30.0
Quirbach et al. 2009 [42]	*Skeletal Radiol*	Retrospective	19.8	Talus	MACT	12	33.3	32.8
Rosa et al. 2015 [43]	*J Orthop Traumatol*	Retrospective	148.0	Knee	pACI	15	40.0	21.0
Sadlik et al. 2017 [44]	*Foot Ankle Surg*	Retrospective	46.4	Talus	OAT	10	40.0	37.0
Schneider et al. 2016 [45]	*J Orthop Surg*	Prospective, Randomised	12.0	Knee	MFX	13	50.0	47.0
				Knee	MFX	4	50.0	37.0
Schüttler et al. 2019 [46]	*Arch Orthop Trauma Surg*	Prospective	60.0	Knee	mACI	23	34.0	
Siebold et al. 2018 [47]	*Knee Surg Sports Traumatol Arthrosc*	Prospective	34.8	Knee	mACI	30	36.0	36.0
Shimozono et al. 2018 [48]	*Am J Sports Med*	Retrospective	52.0	Talus	OAT	63	42.9	36.0
			45.0	Talus	OAT	31	32.3	34.0
Shimozono et al. 2018 [49]	*Bone Joint Surg*	Retrospective	26.3	Talus	OAT	25	64.0	38.4
			22.3	Talus	OAT	16	37.5	43.6
Usuelli et al. 2018 [50]	*Knee Surg Sports Traumatol Arthrosc*	Retrospective	24.0	Talus	AMIC	20	45.0	36.1
Valderrabano et al. 2013 [51]	*Am J Sports Med*	Retrospective	30.9	Talus	AMIC	26	30.8	34.6
Weigelt et al. 2019 [52]	*Am J Sports Med*	Retrospective	56.4	Talus	AMIC	33	4.2	35.1
Wiewiorski et al. 2013 [53]	*Clin Radiology*	Retrospective	23.3	Talus	AMIC	23	30.4	34.2

### Outcomes of interest

The MOCART score at last follow-up evidenced no association with patients’ age (*P* = 0.6), sex (*P* = 0.1), BMI (*P* = 0.06), defect size (*P* = 0.9) or prior length of symptoms (*P* = 0.9). For chondral defects of the knee, no association was found between the MOCART score and IKDC (*P* = 0.9), the Lysholm Knee Scoring Scale (*P* = 0.2), Tegner Activity Scale (*P* = 0.2), VAS (*P* = 0.07), rate of failure (*P* = 0.2) and revision (*P* = 0.9). For chondral defect of the talus, no significant associations were found between the MOCART score and the AOFAS (*P* = 0.3), Tegner Activity Scale (*P* = 0.4), VAS (*P* = 0.1), rate of failure (*P* = 0.1) and revision (*P* = 0.7) (Table 2).

**Table 2:** Overall results of the analyses

Endpoint	*r*	*P*
Patient characteristics		
Sex	−0.22	0.1
Mean age	0.08	0.6
BMI	−0.54	0.06
Defect size (cm^2^)	−0.02	0.9
Prior symptoms (months)	−0.01	0.9
Knee chondral defects		
VAS	−0.45	0.07
Tegner	−0.55	0.2
Lysholm	−0.71	0.2
IKD	0.00	0.9
Rate of failure	−0.30	0.2
Rate of revision	0.01	0.9
Talus chondral defects		
VAS	−0.33	0.1
Tegner	−0.24	0.4
AOFAS	0.29	0.3
Rate of failure	−0.44	0.1
Rate of revision	0.10	0.7

## Discussion

According to the main findings of this systematic review, the MOCART score showed no evidence of a statistically significant association with patient characteristics and surgical outcome in patients who underwent surgical management for knee and talus OL.

The MOCART score did not correlate with any of the other considered scores, namely VAS, AOFAS, IKD, Tegner and Lysholm. This finding corroborates the hypothesis that the MOCART is not a reliable tool for clinical assessment in the setting of osteochondral lesions of the knee and talus, as it does not reflect or associates to any other clinical finding. Similar results were obtained by Casari et al., who did not observe correlations between the MOCART score and other variables such as age, AOFAS score and Tegner score in patients with OL of the talus [2]. While a correlation between VAS and/or the knee injury and osteoarthritis outcome score (KOOS) and different items of the MOCART score was observed in a previous study [17], in particular ‘filling the defect’, ‘structure’ and ‘subchondral bone’ [17], no evaluation of the MOCART score as a whole and VAS or KOOS was performed in that study. In addition, the overall MOCART score was not associated with the trabecular bone parameters in the injured and contralateral knee [18].

Notably, we did not observe any association between the MOCART score and the rate of failure and revision. This finding suggests that the MOCART score does not have a prognostic value in the assessment of the outcomes of surgical management of OL in the knee and talus. Also, the MOCART score did not have sufficient inter-rater reproducibility to allow reliable and univocal use in clinical practice [1].

Three modified versions of the MOCART score are presently available, namely the MOCART 2.0 [19], the modified MOCART [20] and the MOCART 3D [10]. The modified MOCART showed inhomogeneous inter-class correlation coefficients, and no strong correlation could be established between the score and the arthroscopic findings in OL of the talus [20]. The MOCART 2.0 also did not show any association with clinical parameters [2]. On the other hand, however, many items of the MOCART and MOCART 3D showed good correlations with histological scoring systems [10]. Given these limitations, it is difficult to interpret the lack of relationship between age, sex, BMI and defect size and the MOCART score. One possible key is that the MOCART score is not influenced by these factors, or that age, sex and BMI do not influence the extent of OL lesions. The available data do not allow clarification of this issue.

Overall, the question arises whether MRI is a reliable enough tool to assess chondral damage and to follow-up OL treatment, or whether the MOCART score is not a powerful enough tool to allow association of imaging and clinical data. Numerous studies support the use of MRI when following patients with OL of the knee and of the talus. MRI changes of the chondral surface correlate with the structure of the underlying trabecular bone [18], and the combination of clinical parameters with specific MR imaging acquisition represents a promising tool in the follow-up of OL treatment [4, 10]. These findings, combined with the findings of a present study, support the hypothesis that the MOCART score, more than the MRI itself, is not a reliable tool in the follow-up of patients who underwent surgical treatment of OL lesions. However, strong evidence supporting the use of MRI as gold standard in the assessment of OL and its treatment is still lacking [7].

This study does not come without limitations. First, only the evaluation of the MOCART score and not of its successive implementation was possible given the lack of data available for the analysis. Although several studies focused on the management of chondral defects of the knee and talus, relevant quantitative data available for inclusion and analysis were limited. The lack of RCTs also represent an inevitable bias in the interpretation of the results.

## Conclusion

The MOCART score demonstrated no association with patient characteristics and surgical outcome in patients who underwent surgical management for chondral defects of the knee and talus.

## Data Availability

The data underlying this article are available in the article and in its online supplementary material.

## Abbreviations

MOCART: Magnetic Resonance Observation of Cartilage Repair Tissue
VAS: Visual analogue scale
OL: Osteochondral lesions
MRI: Magnetic resonance imaging
PRISMA: Preferred Reporting Items for Systematic Reviews and Meta-Analyses
AOFAS: American Orthopedic Foot and Ankle Score
IKDC: International Knee Documentation Committee
BMI: Body mass index
